# Efficacy and safety of IV/PO moxifloxacin and IV piperacillin/tazobactam followed by PO amoxicillin/clavulanic acid in the treatment of diabetic foot infections: results of the RELIEF study

**DOI:** 10.1007/s15010-012-0367-x

**Published:** 2012-11-23

**Authors:** N. C. Schaper, M. Dryden, P. Kujath, D. Nathwani, P. Arvis, P. Reimnitz, J. Alder, I. C. Gyssens

**Affiliations:** 1 Department of Internal Medicine, Division of Endocrinology, CARIM and CAPHRI Institute, Maastricht University Medical Center+, P.O. Box 5800, 6202 AZ Maastricht, The Netherlands; 2 Royal Hampshire County Hospital, Winchester, Hampshire UK; 3 University Clinic of Luebeck, Ratzeburger Allee 160, Luebeck, Germany; 4 Infection Unit, Ninewells Hospital and Medical School, Dundee, UK; 5 Bayer HealthCare, Loos, France; 6 Bayer Pharma AG, Wuppertal, Germany; 7 Bayer HealthCare, Montville, NJ USA; 8 Department of Medicine, Nijmegen Institute for Infection, Inflammation and Immunity (N4i), Radboud University Nijmegen Medical Centre, Nijmegen, The Netherlands; 9 Department of Medical Microbiology and Infectious Diseases, Canisius Wilhelmina Hospital, Nijmegen, The Netherlands; 10 Hasselt University, Diepenbeek, Belgium

**Keywords:** Antibiotic therapy, Diabetic foot infection, Fluoroquinolone, Moxifloxacin, Controlled clinical trial, Sequential therapy, Diabetic foot ulcer

## Abstract

**Objective:**

The aim was to compare the efficacy and safety of two antibiotic regimens in patients with diabetic foot infections (DFIs).

**Methods:**

Data of a subset of patients enrolled in the RELIEF trial with DFIs requiring surgery and antibiotics were evaluated retrospectively. DFI was diagnosed on the basis of the modified Wagner, University of Texas, and PEDIS classification systems. Patients were randomized to receive either intravenous/oral moxifloxacin (MXF, *N* = 110) 400 mg q.d. or intravenous piperacillin/tazobactam 4.0/0.5 g t.d.s. followed by oral amoxicillin/clavulanate 875/125 mg b.d. (PIP/TAZ–AMC, *N* = 96), for 7–21 days until the end of treatment (EOT). The primary endpoint was clinical cure rates in the per-protocol (PP) population at the test-of-cure visit (TOC, 14–28 days after EOT).

**Results:**

There were no significant differences between the demographic characteristics of PP patients in either treatment group. At TOC, MXF and PIP/TAZ–AMC had similar efficacy in both the PP and intent-to-treat (ITT) populations: MXF: 76.4 % versus PIP/TAZ–AMC: 78.1 %; 95 % confidence interval (CI) −14.5 %, 9.0 % in the PP population; MXF: 69.9 % versus PIP/TAZ–AMC: 69.1 %; 95 % CI −12.4 %, 12.1 % in the ITT population. The overall bacteriological success rates were similar in both treatment groups (MXF: 71.7 % versus PIP/TAZ–AMC: 71.8 %; 95 % CI −16.9 %, 10.7 %). A similar proportion of patients (ITT population) experienced any adverse events in both treatment groups (MXF: 30.9 % versus PIP/TAZ–AMC: 31.8 %, respectively). Death occurred in three MXF-treated patients and one PIP/TAZ–AMC-treated patient; these were unrelated to the study drugs.

**Conclusion:**

Moxifloxacin has shown favorable safety and efficacy profiles in DFI patients and could be an alternative antibiotic therapy in the management of DFI. Clinical trial: NCT00402727.

## Introduction

Diabetic foot infections (DFIs) are the most common cause of hospitalization in people with diabetes, accounting for more hospital bed days than any other diabetic complication [1]. DFIs have a high burden of disease, for both the patient and the health care system. Unless promptly treated, clinical outcomes are poor, leading to impaired wound healing in case of a diabetic foot ulcer, possible amputation of the lower extremity, and significant financial costs [2, 3]. Diabetic patients with a DFI are around 50 times more likely to be hospitalized and 150 times more likely to undergo lower extremity amputation than diabetic patients without foot infections [3, 4]. It has been estimated that around 1 million patients with diabetes undergo limb amputation each year, a surgical procedure that is associated with significant morbidity and mortality, as well as major social, psychological, and financial consequences [5, 6].

Peripheral arterial disease (PAD) is common in patients with DFI, and is an important predictor of outcome [7]. For this reason, the Infectious Diseases Society of America (IDSA) has recommended the inclusion of PAD patients in order to improve the quality of clinical trials evaluating antimicrobial therapy in skin and soft tissue infection. Despite this recommendation, however, few patients with PAD have been included in DFI trials to date [8] and outcome data in this population are lacking.

The most important pathogens causing DFIs are the aerobic Gram-positive cocci (e.g., *Staphylococcus aureus*), Gram-negative aerobes (e.g., *Enterobacteriaceae*), and anaerobic pathogens (e.g., *Bacteroides* spp.) [3]. Most mild to moderate infections are caused by Gram-positive bacteria, while more severe or chronic infections are usually polymicrobial [3] and almost half can include anaerobes [9]. The severity of DFIs has been classified broadly as mild, moderate, or severe. However, a lack of consensus and consistency in defining infection severity makes comparison across published studies complicated. Although various systems have been developed, no single system has found universal acceptance. The most established system of classification of ulceration, the Wagner system [10], has been employed for more than 25 years, but does not apply to infection severity, assessing only ulcer depth and the presence of osteomyelitis or gangrene. While the University of Texas diabetic wound classification system has been validated in DFI and includes the presence of infection, as well as ulcer depth and ischemia, infection severity is, again, not assessed [11, 12]. At a more recent international consensus conference, a system of classification of diabetic foot ulcers [perfusion, extent/size, depth/tissue loss, infection, and sensation (PEDIS)] was developed that includes all the key elements of severity of infection. The newer system has been adopted by the IDSA and has been validated by Lavery et al. [13–15].

Optimal management of DFIs is multimodal, involving surgical debridement, antibiotic therapy, scrupulous wound care and offloading, glycemic control, and assessment and treatment of underlying vascular disease if lower extremity amputations are to be avoided [2]. In patients with deep ulcers, immediate drainage with removal of all the necrotic tissue should be considered and intravenous antibiotics are started after material is obtained for culture. In the absence of adequate microbiological information, the challenge for clinicians is to select an appropriate empirical antibiotic regimen because timely and proper management of diabetic foot infection together with surgery [14] is key to prevent further complications. The patient’s previous antibiotic exposure, the pathogens most commonly isolated, and local rates of resistance should all be considered.

Fluoroquinolones, such as moxifloxacin (MXF), offer possible advantages over other classes of antimicrobial agents for the empirical treatment of DFIs, due to their broad spectrum of activity and pharmacodynamic properties [9, 16]. MXF, in particular, has an extended spectrum of activity against aerobic and anaerobic pathogens [9, 16] and can be given intravenously (IV) or orally (PO). The switch between the two formulations is simple as they have similar pharmacokinetic properties [17], and MXF penetrates well into inflamed subcutaneous tissues [18–20]. A previous IV/PO study of MXF versus β-lactam/β-lactamase inhibitor showed MXF to be effective and well tolerated in the treatment of complicated skin and skin structure infections (cSSSIs) [21].

The rationale of the RELIEF study was to provide additional data on the efficacy and safety of MXF in four specific cSSSI diagnoses (major abscess, DFI, wound infection, infected ischemic ulcer) [22]; the present paper reports on the efficacy and safety of MXF versus a β-lactam/β-lactamase inhibitor combination in the subgroup of patients with DFI. The RELIEF study is unique in that it addresses the limitations of previous studies by stratification at inclusion, according to the severity of cSSSI and baseline surgery. Moreover, in contrast to most earlier DFI studies, patients with PAD were included. The combination of a β-lactam antibiotic and a β-lactamase inhibitor (piperacillin/tazobactam) was chosen as the comparator, as this combination is known to be effective in treating cSSSIs [23].

## Methods

### Study design and patients

The RELIEF study was a prospective, randomized, double-dummy, double-blind, multinational, multicenter study [22], involving adult men and women (≥18 years old) with a diagnosis of complicated bacterial skin and skin structure infection of <21 days duration that required hospitalization and initial parenteral antibiotic treatment for ≥48 h. DFI patients were enrolled between 11th October 2006 and 9th June 2008. Diagnoses were validated by an independent Data Review Committee (DRC). All patients were stratified before randomization according to the severity of illness (risk class I and II, or III and IV) using the Wilson scoring system [24].

Patients had to have at least one of the following criteria: involvement of deep soft tissue (e.g., fascia, muscle layer), need for significant surgical intervention (e.g., surgical drainage or drainage procedure guided by imaging and/or debridement), or association with a significant underlying disease that could complicate treatment response (e.g., cancer except basal- or squamous-cell skin cancers, cardiac, hepatic, immunological, renal, respiratory, transplantation, or vascular disease). Amputation of part of the foot could have been performed in patients who had extensive infection and would not have benefited from other surgeries, but patients could only be included if a cSSSI was still present after completion of this surgery. All initial surgeries had to be completed within 48 h after beginning infusion of the antibiotics.

All diabetic patients had to have a DFI of mild to severe infection intensity (PEDIS grade 2–4) during randomization with or without osteomyelitis (patients with osteomyelitis could only be enrolled if the infected bone was removed and if residual infection was still present). In order to facilitate the comparison of this study with other recent studies, all ulcers were also categorized according to the University of Texas classification system [11]. As initial (emergency) surgery, including amputation of part of the foot, within 48 h after inclusion was part of the immediate treatment, post-amputation PEDIS infection scores were retrospectively calculated for all patients to confirm that the infection was still requiring antibiotic treatment.

In all patients, glycemic control was measured by the plasma level of glycated hemoglobin (HbA_1c_) values. To describe the severity of infection, body temperature and levels of inflammatory markers including C-reactive protein (CRP), procalcitonin (PCT), and white blood cell count (WBC), were documented. Furthermore, wound depth and type of wound care undertaken were also registered. The presence of peripheral neuropathy (assessed by both vibration perception and light pressure testing) and PAD [defined as ankle–brachial index (ABI) <0.9 and/or foot pulses barely or not palpable] were also recorded.

Patients who had received therapy with a systemic or topical antimicrobial for >24 h in the previous 7 days were excluded from the study, unless they were considered to be clinical failures (i.e., the patient showed no response or had worsening of clinical signs and symptoms despite treatment for ≥3 days) and a culture before enrolment showed persistence of a pathogen that was susceptible to the study drugs.

### Study treatments

Patients received sequential IV/PO MXF, 400 mg q.d., plus matching placebo or IV piperacillin/tazobactam (PIP/TAZ), 4.0/0.5 g t.d.s., followed by PO amoxicillin/clavulanic acid (AMC) 875/125 mg b.d. They were treated for a minimum of 7 and a maximum of 21 days. The switch from IV to PO drug was at the investigator’s discretion, provided the patient was improving on IV therapy, had no fever, that gastrointestinal absorption was adequate, and the patient had received IV study drug for at least 48 h. The total duration of therapy was also at the investigator’s discretion.

### Assessments

Clinical efficacy was assessed during treatment (days 3–5), at the end of treatment (EOT, 7–21 days after inclusion), and at the test-of-cure visit (TOC, 14–28 days after EOT). The primary efficacy variable was clinical response at TOC as assessed by the independent DRC. Photographs of skin lesions were taken at each assessment and were used by the DRC, together with blinded patient data, to evaluate clinical response to the study drug at TOC. Patients were considered as clinical failures at any visit who had insufficient lessening of the clinical signs and symptoms of infection such that additional or alternative antimicrobial therapy was required (with or without additional surgery). Clinical cures/successes were patients considered to be cured at TOC and who were not considered as failures at EOT. Patients whose outcome was considered as clinical failure at EOT were counted in the total number of clinical failures at TOC. Safety assessment was based on physical examination, vital signs, ECG, adverse events (AEs), and standard laboratory tests throughout the study.

### Patient populations

The RELIEF study had a non-inferiority design [22] and the current analysis is based on the subpopulation of patients with DFI in the RELIEF study [25]. The main analysis population was the per-protocol (PP) population, consisting of all patients who had received the study drug for ≥72 h (in case of clinical failure) or 7 full days (in case of success), had received ≥48 h of IV therapy, had documented compliance of ≥80 % with study medication, had no protocol violations influencing treatment efficacy, and for whom a clinical evaluation at TOC other than ‘indeterminate’ (subjects in whom a clinical assessment could not be determined as improvement, resolution, or failure) was available. Confirmatory and safety analyses were carried out on the intent-to-treat (ITT) population (all randomized patients who received at least one dose of study drug and had at least one observation after taking study medication).

Bacteriological evaluations were performed using the microbiologically valid (MBV) population (all PP patients for whom at least one causative organism could be cultured from an appropriate specimen within 48 h before or following randomization and a bacteriological evaluation at TOC other than ‘indeterminate’ was available). Confirmatory analyses were carried out on the ITT with organisms population, which included all patients in the ITT population for whom at least one causative organism could be cultured from an appropriate specimen within 48 h before or following randomization.

### Bacteriological assessments

Bacteriological evaluation was performed at enrolment, during therapy (days 3–5), at EOT, and at TOC. Biospecimens were taken by needle aspiration, tissue biopsy, or curettage for culture, identification of bacteria, and for susceptibility testing against a variety of antibiotics, including study drugs. In addition, susceptibility to oxacillin was tested when staphylococci were isolated and extended-spectrum beta-lactamase (ESBL) activity was determined for *Enterobacteriaceae* species. Acceptable culture specimens included skin biopsy, curettage of the wound base after debridement, tissue or bone biopsy, aspiration of purulent secretions, or a leading-edge needle aspiration for subjects with cellulitis. Cultures should not have been obtained using a swab. All cultured organisms underwent genus and species identification at the central microbiology laboratory. In subjects with initial baseline-positive blood cultures, blood cultures were repeated until the result was negative.

The study microbiologist reviewed all information before un-blinding the patients according to whether bacteria were colonizing in nature, whether they were part of the normal flora, or whether they likely played a pathogenic role based on its nature and quantitative culture results. Criteria indicating infection rather than colonization included growth of the organism from the deep tissue in the setting of inflammation and purulent drainage [14] and Gram-stained smear of the wound showing neutrophils and organisms morphologically compatible with those grown in culture.

### Statistical methods

Treatment groups were compared using the Mantel–Haenszel estimates and 95 % confidence intervals (CIs) for differences in the cure rates at TOC. For the PP analyses of the clinical and bacteriological responses, the treatment comparisons were performed as “success” versus “non-success” (missing or indeterminate outcomes were not allowed in the PP population). For the ITT analyses, a comparison of “success” versus “non-success” was performed; additionally, an analysis of “success” versus “failure” was compared. Statistical analyses were stratified by the severity of illness based on the Wilson scoring system and the presence or absence of a cSSSI-related surgical procedure prior to or scheduled to take place within 48 h of study entry. Non-inferiority of MXF was demonstrated if the lower limit of the 95 % CI was above −10 %. As the RELIEF study was not powered to look at subgroups, the 95 % CIs presented here are exploratory in nature. Adverse events were classified according to MedDRA code and their severity and relationship to study drug assessment. Vital signs and laboratory data were analyzed descriptively.

## Results

### Patient disposition

**Fig. 1:**
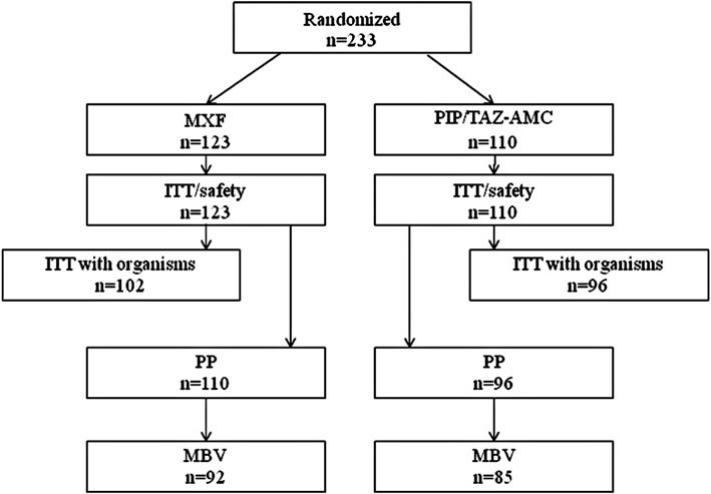
Disposition of patients with diabetic foot infection (DFI). *ITT* intent-to-treat, *MBV* microbiologically valid, *MXF* moxifloxacin, *PIP/TAZ–AMC* piperacillin/tazobactam–amoxicillin/clavulanic acid, *PP* per-protocol

A total of 233 patients with a DFI were randomized into the study (ITT population), of whom 206 were valid for the PP analysis (MXF = 110, PIP/TAZ–AMC = 96). The patient disposition is shown in Fig. 1. There were no significant differences between the demographic characteristics of PP patients in either treatment group (Table 1), except that there were more men in the PIP/TAZ–AMC group and the mean glycosylated hemoglobin (HbA_1c_) levels were higher in the MXF arm. Patients in both study arms were overweight, had elevated CRP and PCT levels, and more than 80 % were febrile. HbA_1c_ levels were considerably higher than those observed in the overall RELIEF population (7.0–7.4 %) [22], but are consistent with other studies in patients with DFI [26, 27]. PAD was evident in over 65 % of all patients, out of whom 129 (62.6 %) had barely or non-palpable pulses in both dorsalis pedis and posterior tibialis arteries, and almost half had peripheral neuropathy (Table 1). Most infection types (>87 %) were community acquired (CA). At baseline, most patients had moderate to severe DFIs, with a PEDIS infection score of 3 or 4. Initial surgeries (most commonly amputation or extensive debridement) were carried out on 150 patients (MXF: 70.9 % versus PIP/TAZ: 75.0 %). Although the baseline pre-amputation PEDIS infection scores were similar in the two groups of patients (Table 1), it was observed that, numerically, but not statistically significantly, more patients in the MXF arm had an amputation as initial (<48 h after randomization) surgery (MXF: *n* = 51, 46.4 % and PIP/TAZ–AMC: *n* = 33, 34.3 %). PEDIS infection scores were calculated post-amputation to assess whether this had introduced a bias. More patients in the MXF arm had a calculated PEDIS score of 3 (*n* = 29, 56.9 %) than in the PIP/TAZ–AMC arm (*n* = 13, 39.4 %) post-amputation. A similar number of patients in each treatment arm had mild infection, i.e., PEDIS score of 2 (MXF: *n* = 22, 43.1 % and PIP/TAZ–AMC: *n* = 20, 60.6 %), while none had a severe infection (i.e., PEDIS score of 4) following amputation in either arm. The mean [standard deviation (SD)] duration of combined IV/PO therapy was similar, being 14.5 (4.5) days in the MXF and 14.2 (4.4) days in the PIP/TAZ–AMC groups, respectively. There was no significant difference between the two arms regarding the length of either PO or IV treatments. Intravenous infusion lasted for 8.1 ± 4.1 days in the MXF group and 7.5 ± 3.3 days in the PIP/TAZ–AMC group; PO administration of drugs lasted for 8.1 ± 3.2 days in the MXF group and 8.4 ± 3.1 days in the PIP/TAZ–AMC group. The number of patients who received other antibiotics before treatment with study drugs was similarly low in the MXF (8.2 %) and PIP/TAZ–AMC (8.3 %) treatment groups, respectively.

**Table 1:** Demographic and disease characteristics of patients at baseline with diabetic foot infection (DFI) in the RELIEF study [per-protocol (PP) population]

	MXF (*N* = 110)	PIP/TAZ–AMC (*N* = 96)
Sex, male, *n* (%)	61 (55.5)	69 (71.9)
Mean age (years) (SD)	58.9 (10.2)	59.5 (10.1)
Mean BMI, kg/m^2^ (SD)	28.9 (5.7)	28.6 (4.7)
Temperature >38 °C, *n* (%)	98 (89.1)	79 (82.3)
Mean WBC, 10^9^/L (SD)	10.0 (4.0)	9.3 (3.8)
Mean HbA_1c_ (%) (SD)	9.7 (2.5)	9.0 (2.1)
Mean CRP, mg/L (SD)	8.3 (8.8)	8.7 (8.4)
Mean PCT, ng/ml (SD)	0.2 (0.3)	0.2 (0.6)
Peripheral neuropathy, *n* (%)		
Vibration perception test—negative^c^	44 (41.5)	48 (51.6)
Light pressure test (plantar surface of heel)—negative^d^	52 (49.5)	44 (47.8)
Peripheral arterial disease^a^, *n* (%)	72 (65.5)	68 (70.8)
ABI <0.9	46 (41.8)	42 (43.8)
Absent or barely palpable dorsalis pedis and posterior tibialis pulses	66 (60.0)	63 (65.6)
Infection type, *n* (%)		
Community acquired	96 (87.3)	87 (90.6)
Hospital acquired	14 (12.7)	9 (9.4)
Mean time since occurrence of symptoms (days) (SD)	9.5 (5.4)	9.2 (5.6)
Pre-therapy antibiotic use, *n* (%)	9 (8.2)	8 (8.3)
Mean lesion area (cm^2^) (SD)	46.9 (66.4)	35.1 (48.5)
Deepest tissue layer infected, *n* (%)		
Dermis	10 (9.1)	6 (6.3)
Subcutaneous fat	12 (10.9)	4 (4.2)
Fascia, muscle, or deeper	88 (80.0)	86 (89.6)
Type of surgery during first 48 h^b^, *n* (%)		
No surgery	32 (29.1)	24 (25.0)
Abscess drainage	28 (25.5)	31 (32.3)
Local debridement	21 (19.1)	17 (17.7)
Extensive debridement	32 (29.1)	38 (39.6)
Primary closure	12 (10.9)	8 (8.3)
Amputation	51 (46.4)	33 (34.4)
Graft surgery	0 (–)	1 (1.0)
Removal of infected bone area	21 (19.1)	19 (19.8)
Revascularization	1 (0.9)	1 (1.0)
Necrectomy	0 (–)	1 (1.0)
University of Texas wound classification^e^, *n* (%)		
Grade 0, Infected	0 (–)	1 (1.1)
Grade 0, Ischemic^a^	1 (0.9)	0 (–)
Grade I, Infected	4 (3.7)	1 (1.1)
Grade I, Ischemic^a^	11 (10.3)	8 (8.5)
Grade II, Infected	16 (15.0)	14 (14.9)
Grade II, Ischemic^a^	45 (42.1)	43 (45.7)
Grade III, Infected	9 (8.4)	2 (2.1)
Grade III, Ischemic^a^	21 (19.6)	25 (26.6)
Wilson score, mean (SD)	100.6 (21.9)	103.5 (22.5)
Risk class I, *n* (%)	5 (4.5)	4 (4.2)
Risk class II, *n* (%)	20 (18.2)	8 (8.3)
Risk class III, *n* (%)	34 (30.9)	33 (34.4)
Risk class IV, *n* (%)	51 (46.4)	51 (53.1)
Baseline PEDIS infection score classification of all patients^e^, *n* (%)		
2 (Mild)	14 (13.1)	8 (8.5)
3 (Moderate)	87 (81.3)	81 (86.2)
4 (Severe)	6 (5.6)	5 (5.3)
Baseline PEDIS infection score classification before amputation^f^, *n* (%)		
2 (Mild)	1 (2.0)	0 (0.0)
3 (Moderate)	47 (92.2)	31 (93.9)
4 (Severe)	3 (5.9)	2 (6.1)

*ABI* ankle–brachial index; *BMI* body mass index; *CRP* C-reactive protein; *HbA*_*1c*_ glycosylated hemoglobin; *MXF* moxifloxacin; *PCT* procalcitonin, *PEDIS* perfusion, extent/size, depth/tissue loss, infection, and sensation; *PIP/TAZ–AMC* piperacillin/tazobactam–amoxicillin/clavulanic acid; *SD* standard deviation; *WBC* white blood cell count

^a^Defined as ABI <0.9 and/or foot pulses barely or not palpable; foot pulses as barely or not palpable were examined in the dorsalis pedis and posterior tibialis arteries

^b^Patients could have ≥1 surgical procedure

^c^ *n* = 106 MXF, *n* = 93 PIP/TAZ–AMC

^d^ *n* = 105 MXF, *n* = 92 PIP/TAZ–AMC

^e^ *n* = 107 MXF, *n* = 94 PIP/TAZ–AMC

^f^Patients (*n* = 51 MXF, *n* = 33 PIP/TAZ–AMC) exclusively with amputation as initial surgery (either prior to enrolment or within 48 h after the start of study medication)

### Baseline bacteriology

Particular attention was paid as to whether the organisms isolated represented colonizers or pathogens. The presented data in this paper refer to pathogenic bacteria. A total of 92 MXF-treated and 85 PIP/TAZ–AMC-treated MBV patients had 347 pathogenic organisms isolated at baseline (MXF: 177, PIP/TAZ–AMC: 170), with similar microbiological profiles seen across the two treatment groups. The most commonly isolated organisms as a percentage of all organisms isolated were: methicillin-susceptible *Staphylococcus aureus* (MSSA) (MXF: 53, 29.9 %; PIP/TAZ–AMC: 57, 33.5 %), *Enterococcus faecalis* (MXF: 30, 16.9 %; PIP/TAZ–AMC: 29, 17.1 %), methicillin-resistant *S. aureus* (MRSA) (MXF: 11, 6.2 %; PIP/TAZ–AMC: 12, 7.1 %), non-ESBL-producing *Escherichia coli* (MXF: 8, 4.5 %; PIP/TAZ–AMC: 11, 6.5 %), *Bacteroides fragilis* (MXF: 3, 1.7 %; PIP/TAZ–AMC: 4, 2.4 %), *Streptococcus pyogenes* (MXF: 3, 1.7 %; PIP/TAZ–AMC: 2, 1.2 %), and ESBL-producing *E. coli* (MXF: 1, 0.6 %; PIP/TAZ–AMC: 1, 0.6 %). Polymicrobial infections were common, occurring in more than half of all patients (MXF: 60.9 %; PIP/TAZ–AMC: 62.3 %).

### Clinical efficacy

**Fig. 2:**
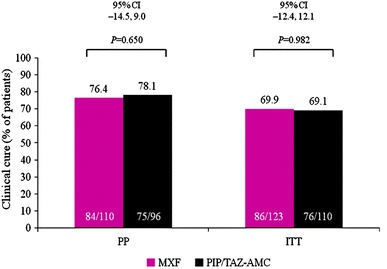
Clinical cure at the test-of-cure visit (TOC) in the per-protocol (PP) and intent-to-treat (ITT) patient populations. *CI* confidence interval, *ITT* intent-to-treat, *MXF* moxifloxacin, *PIP/TAZ–AMC* piperacillin/tazobactam–amoxicillin/clavulanic acid, *PP* per-protocol

Clinical cure rates were similar between treatment groups (Fig. 2). At TOC, MXF and PIP/TAZ–AMC had similar efficacy in both the PP and ITT populations (MXF: 76.4 % versus PIP/TAZ–AMC: 78.1 %; 95 % CI −14.5 %, 9.0 % in the PP population; MXF: 69.9 % versus PIP/TAZ–AMC: 69.1 %; 95 % CI −12.4 %, 12.1 % in the ITT population). Similar results were seen in patients of the MBV population (MXF: 69/92, 75.0 % versus PIP/TAZ–AMC: 64/85, 75.3 %; 95 % CI −15.8 %, 10.6 %) and of the ITT with organisms population (MXF: 71/102, 69.6 % versus PIP/TAZ–AMC: 65/96, 67.7 %; 95 % CI −13.0 %, 14.1 %). Good clinical efficacy was seen across the range of infection severities, as measured by the University of Texas wound classification, baseline PEDIS infection scores, or the Wilson classification system (Table 2).

**Table 2:** Clinical success by disease severity scoring system (per-protocol population)

	MXF^b^, *n*/*N* (%)	PIP/TAZ–AMC^b^, *n*/*N* (%)
Texas wound classification		
Grade 0	0/1 (0)	1/1 (100)
Infected		1/1 (100)
Ischemic^a^	0/1 (0)	
Grade I	11/15 (73.3)	7/9 (77.8)
Infected	3/4 (75.0)	1/1 (100)
Ischemic^a^	8/11 (72.7)	6/8 (75.0)
Grade II	45/61 (73.8)	47/57 (82.5)
Infected	12/16 (75.0)	14/14 (100)
Ischemic^a^	33/45 (73.3)	33/43 (76.7)
Grade III	25/30 (83.3)	18/27 (66.7)
Infected	9/9 (100)	2/2 (100)
Ischemic^a^	16/21 (76.2)	16/25 (64.0)
PEDIS infection score classification prior to any surgery		
2 (Mild)	12/14 (85.7)	6/8 (75.0)
3 (Moderate)	66/87 (75.9)	64/81 (79.0)
4 (Severe)	3/6 (50.0)	3/5 (60.0)
Wilson classification		
Risk class I	4/5 (80.0)	4/4 (100)
Risk class II	15/20 (75.0)	7/8 (87.5)
Risk class III	30/34 (88.2)	28/33 (84.8)
Risk class IV	35/51 (68.6)	36/51 (70.6)

*MXF* moxifloxacin, *PIP/TAZ–AMC* piperacillin/tazobactam–amoxicillin/clavulanic acid

*n*/*N* = number of patients with clinical cure/total number of patients

*P* > 0.05 in all cases, Cochran–Mantel–Haenszel test

^a^Defined as ABI <0.9 and/or foot pulses barely or not palpable

^b^MXF: *N* = 107; PIP/TAZ–AMC: *N* = 94

Additional surgeries >48 h after the start of therapy (including amputation) were carried out on 20.9 % of MXF-treated and 25.0 % of PIP/TAZ–AMC-treated patients (PP populations). Of these, fewer MXF versus PIP/TAZ–AMC patients required amputation (8.2 versus 16.7 %, respectively), but these differences were not statistically different.

### Bacteriological response

The susceptibility to the administered antibiotics of the most common bacteria isolated in our DFI patients is shown in Table 3. The most prevalent causative species (MSSA) was susceptible to both antibiotics used, while the second most frequently isolated species (*E. faecalis*) was resistant to both MXF and PIP/TAZ in a small percentage of cases [i.e., 5 out of 34 isolates (14.7 %) and 5 out of 33 isolates (15.1 %) in both treatment groups, respectively].

**Table 3:** Pre-therapy minimum inhibitory concentrations of study drugs against common pathogens (ITT with pathogens population)

	*N*	MXF (mg/L)			PIP/TAZ (mg/L)			AMC (mg/L)		
		MIC_50_	MIC_90_	Range	MIC_50_	MIC_90_	Range	MIC_50_	MIC_90_	Range
*Staphylococcus aureus*										
Methicillin-susceptible	125	0.03	0.06	≤0.015–4.0	1.0	2.0	0.5–2.0	0.50	1.0	≤0.06–2.0
Methicillin-resistant	27	2.0	8.0	0.12–8.0	16.0	>128	4.0 to >128	8.0	>32.0	2.0 to >32.0
*Enterococcus faecalis*	67	0.25	16.0	0.12–16.0	4.0	8.0	2.0 to >128	1.0	1.0	0.25–32.0
*Escherichia coli*										
Non-ESBL-producing	20	0.03	32.0	0.03 to >32.0	2.0	2.0	0.5–2.0	4.0	8.0	2.0–8.0
*Bacteroides fragilis*	10	0.5	2.0	0.5–4.0	0.25	0.5	0.12–1.0	–	–	–

*ESBL* extended-spectrum beta-lactamase, *N* total number of isolates, *ITT* intent-to-treat, *MXF* moxifloxacin, *PIP/TAZ–AMC* piperacillin/tazobactam–amoxicillin/clavulanic acid; *Streptococcus pyogenes* (*n* = 6): for MXF: MIC_50_ 0.12, MIC_90_ 0.25, range 0.12–0.25; for PIP/TAZ: MIC_50_ ≤0.25, MIC_90_ ≤0.25, range ≤0.25 to ≤0.25; for AMC: MIC_50_ ≤0.06, MIC_90_ ≤0.06, range ≤0.06 to ≤0.06; *Escherichia coli* ESBL-producing (*n* = 3): for MXF: MIC_90_ ≥32.0; for PIP/TAZ: MIC_90_ = 16.0; for AMC: MIC_90_ = 16.0

Overall the proportion of patients with bacteriological success in the MBV (Table 4) population were similar across treatment arms (MXF: 71.7 % versus PIP/TAZ–AMC: 71.8 %) and no difference was found between treatment groups (95 % CI −16.9 %, 10.7 %). Data from patients in the ITT with organisms population support that MXF was as effective as PIP/TAZ–AMC (95 % CI −13.0 %, 14.6 %). A similar bacteriological outcome was observed for the most commonly isolated pathogens between the two treatment arms (Table 4). Eradication of MSSA was numerically higher with MXF treatment. In the small number of patients who had MRSA, the proportion achieving eradication for this species was similar. The proportion of patients achieving bacteriological eradication of monomicrobial (MXF: 25/36, 69.4 % versus PIP/TAZ–AMC: 23/32, 71.9 %) or polymicrobial infections (MXF: 43/56, 76.8 % versus PIP/TAZ–AMC: 38/53, 71.7 %) were relatively similar between treatment groups. The lowest percentages (<70 %) of bacteriological success among the most frequent organisms were observed for infections with *E. faecalis* for both treatment groups; this species was mainly isolated from polymicrobial infections.

**Table 4:** Bacteriological success overall and by key organisms

Bacteriological success by patients		
	MXF, *n*/*N* (%)	PIP/TAZ–AMC, *n*/*N* (%)
MBV	66/92 (71.7)	61/85 (71.8)
ITT with organisms	69/102 (67.6)	62/96 (64.6)
Bacteriological success by key organism (MBV population)^a^		
*Staphylococcus aureus*		
Methicillin-susceptible	43/53 (81.1)	39/57 (68.4)
Methicillin-resistant	8/11 (72.7)	10/12 (83.3)
*Streptococcus pyogenes*	3/3 (100)	2/2 (100)
*Enterococcus faecalis*	19/30 (63.3)	20/29 (69.0)
*Escherichia coli*		
ESBL-producing	1/1 (100)	1/1 (100)
Non-ESBL-producing	6/8 (75.0)	8/11 (72.7)
*Bacteroides fragilis*	3/3 (100)	3/4 (75.0)

*ITT* intent-to-treat, *MBV* microbiologically valid, *ESBL* extended-spectrum beta-lactamase, *MXF* moxifloxacin, *PIP/TAZ–AMC* piperacillin/tazobactam–amoxicillin/clavulanic acid

^a^ *n*/*N* = number organisms with eradication or presumed eradication/total number of organisms isolated

The emergence of resistant bacteria with moxifloxacin treatment was rare while on therapy or post-therapy in both treatment groups, and it was only seen for one *Klebsiella pneumoniae* (non-ESBL) isolate (pre-therapy: MIC_90_ = 0.12 mg/L, during therapy: MIC_90_ = 8.0 mg/L, EOT: MIC_90_ = 8.0 mg/L) and one *Acinetobacter baumannii* isolate (pre-therapy: MIC_90_ = 1.0 mg/L, during therapy: MIC_90_ = 2.0 mg/L, EOT: MIC_90_ = 8.0 mg/L).

### Safety

The total numbers of patients with a DFI experiencing any adverse event were comparable between the MXF [38 (30.9 %)] and PIP/TAZ–AMC [35 (31.8 %)] groups (Table 5). Only two moxifloxacin-treated patients (1.6 %) experienced drug-related serious adverse events (SADR) and none (0 %) in the other treatment arm; those two reported SADR cases in the MXF arm occurred as asymptomatic prolongation of ECG QT interval with permanent discontinuation of the study drug. Death occurred in three MXF (2.4 %) and one PIP/TAZ–AMC (0.9 %) treatment patients and none of these was reported to be drug-related (three MXF patients died due to respiratory failure, pulmonary embolism, and cardiovascular and renal failure, and one PIP/TAZ–AMC patient died due to arterial thromboembolism).

**Table 5:** Overview of treatment-emergent adverse events in patients and the most frequent adverse events (≥3 in either treatment group, ITT/safety population)

Event	MXF (*N* = 123), *n* (%)	PIP/TAZ–AMC (*N* = 110), *n* (%)	*P*-value
Adverse event (AE)	38 (30.9)	35 (31.8)	0.89
Diarrhea	1 (0.8)	4 (3.6)	
Gangrene	2 (1.6)	3 (2.7)	
Nausea	2 (1.6)	3 (2.7)	
Blood creatinine increased	3 (2.4)	1 (0.9)	
Creatinine renal clearance decreased	3 (2.4)	1 (0.9)	
Electrocardiogram QT prolonged	3 (2.4)	1 (0.9)	
Pyrexia	1 (0.8)	3 (2.7)	
Abscess limb	0 (–)	3 (2.7)	
Insomnia	3 (2.4)	2 (1.8)	
Hypertension	5 (4.1)	1 (0.9)	
Drug-related AE	12 (9.8)	11 (10.0)	1.00
Premature discontinuation due to AE	5 (4.1)	2 (1.8)	0.45
Serious AE	13 (10.6)	10 (9.1)	0.83
Drug-related SAE	2 (1.6)	0 (0.0)	
Premature discontinuation due to drug-related SAE	2 (1.6)	0 (0.0)	
Deaths	3 (2.4)	1 (0.9)	0.62

*AE* adverse event, *SAE* serious adverse event, *ITT* intent-to-treat, *MXF* moxifloxacin, *PIP/TAZ–AMC* piperacillin/tazobactam–amoxicillin/clavulanic acid

## Discussion

MXF IV/PO therapy had similar efficacy compared with PIP/TAZ–AMC therapy in patients with moderate to severe DFI in a randomized double-blind trial in which patients were prospectively stratified according to the severity of illness and the need for surgery. Previous DFI trials have failed to document important disease parameters and baseline data such as infection severity and wound classification at study entry, making comparison between studies difficult [28–31]. In contrast, the patients in the present study were well characterized, with a range of important baseline characteristics such as body mass index (BMI), degree of glycemic control, levels of inflammatory markers, presence of limb ischemia, wound type and care, and severity of infection being documented. These parameters have been highlighted in a recent review as key parameters to be included in order to improve the quality of future DFI studies [32].

Patients in the current study were recruited across a range of infection severities, though most of the DFI patients (>80 %) had moderate to severe infections with a PEDIS infection score of 3 and could be considered as difficult-to-treat patients. As expected, a relatively high number of patients required initial surgery, one of the cornerstones in the treatment of severe foot infections, which could have introduced bias. However, the post-initial surgery PEDIS infection scores did not differ between both treatment arms.

An additional strength of the current study is that the majority (>65 %) of the RELIEF study patients with a DFI had also signs of PAD. PAD is present in around half of all patients with foot ulcers [7], yet, a number of earlier clinical trials of antibiotics in DFIs enrolled few PAD patients [33, 34] or excluded those with critical limb ischemia [29, 31] or PAD requiring revascularization [23]. In the large-scale Eurodiale study, the outcome of DFIs in patients without PAD was relatively good, but particularly poor results were obtained in DFI patients with PAD [35]. As previously demonstrated, treatment with IV or PO moxifloxacin in DFI patients achieves a concentration well above MIC_90_ for most pathogenic bacteria in peri-necrotic limb tissue [19], suggesting that PK/PD properties of moxifloxacin are able to overcome the potential lack of perfusion due to PAD.

Whether the antibiotic agent reaches its site-of-action with sufficiently high levels is a critical point in DFI patients with PAD and limb ischemia, and has been investigated in previous studies [36, 37]. For example, Zammit et al. [38] have investigated the impact of increasing severity of PAD on the tissue concentration of gentamicin in patients with ischemic ulcers. These authors have found a strong inverse correlation between PAD severity and local limb tissue concentration of the antibiotic drug, suggesting that local blood flow regulation is an important determinant of drug efficacy. Similarly, the penetration of ceftazidime into bone and soft tissues was found to be correlated in another study with the degree of tissue perfusion in both diabetic and non-diabetic patients [34]. Moreover, diminished and impaired microcirculation could well be a complicating factor for why DFI patients require antibiotic treatment for a long period of time (i.e., the mean duration of therapy in the current study was >14 days). There were 17 patients in nine different study centers who had critical limb ischemia with an ABI <0.5 (12 MXF patients and five PIP/TAZ–AMC patients). Only one of these patients underwent a revascularization procedure during the study period, suggesting that the treatment of PAD was in our patients suboptimal and that better results may have been obtained with a more aggressive approach.

The burden of disease due to the DFI was high in our study, with 53 % of all patients undergoing an amputation either as initial treatment or during the course of the treatment. Patients in this study had a severe condition at baseline due to poor glycemic control (mean HbA_1c_ of nearly 10 %) and elevated inflammatory markers (WBC, CRP, and PCT). Earlier diagnosis and treatment of a diabetic foot ulcer with better glycemic control or earlier recognition of the presence of infection in these patients with prompt institution of appropriate multidisciplinary management might have prevented the development of a more severe infection in many of our patients.

In the current study, both MXF and PIP/TAZ–AMC had relatively high clinical efficacy rates with respect to clinical cure at TOC across all patient populations and sequential IV/PO MXF was as effective as IV/PO PIP/TAZ–AMC, thus, confirming the results of previous studies [21, 39].

To date, no single agent has been found to be the most effective for the treatment of moderate to severe DFI and, usually, therapy is selected on an empirical basis, either a broad-spectrum agent or a combination of agents that provide a broad spectrum [21]. The current results with MXF are particularly promising, as not only does MXF offer a broad spectrum of activity, permitting the agent to be used in monotherapy, but its IV and oral formulations are similar in their pharmacokinetic properties, so that the switch between IV and PO is simple.

The range of baseline pathogens isolated and infection types was as anticipated, with the most commonly occurring pathogen being *S. aureus* (approximately 30 %), although the number of isolated anaerobic bacteria was quite low. Polymicrobial infections become more likely with increasing severity and chronicity of the diabetic foot ulcer [40], and these occurred in more than half of our patients. Bacteriological eradication was high in both treatment groups; somewhat higher eradication was seen for MXF versus PIP/TAZ–AMC in polymicrobial infections (76.8 versus 69.8 %). Several previous studies reported higher levels of MRSA infection (30–50 %) [5, 41, 42] than those seen in the RELIEF study (6–7 %). However, the vast majority of the DFIs treated in this study were community-acquired infections, while in most European countries MRSA infections tend to occur more commonly in patients who have been in hospital; moreover, regional differences in MRSA prevalence might also play a role. Nevertheless, MRSA infections are not necessarily more severe than MSSA infections [43]. In this study, MRSA might have been present simply as a colonizer, particularly in the patients in whom MRSA was isolated but who experienced a clinical cure without a specific anti-MRSA agent (notably when there was a low or a lack of susceptibility of MRSA to either MXF or PIP/TAZ–AMC).

Both treatments were well tolerated with similar percentages of patients experiencing any adverse events or drug-related serious adverse events at low frequencies. None of the deaths reported in this clinical trial was drug-related.

Gyssens et al. [22] provide an extensive commentary on the strengths and weaknesses of the RELIEF study design, of which the pre-randomization stratification is identified as a key strength and a feature which has not, to date, been included in the design of other skin and soft tissue infection clinical trials. The strict methodology used in the RELIEF study provided an in-depth and accurate assessment of patients and disease characteristics and assessment by a blinded DRC, prospective use of the Wilson Risk Class, and standardized photo assessment of lesions all helped to remove investigator bias [22]. We acknowledge that subgroup analysis is associated with limitations; in particular, the relatively low number of patients with diabetic foot infections in each treatment arm results in an insufficient statistical power and limits the interpretation of the results of this study to the patient population with DFI in general. Therefore, these results should be interpreted cautiously.

In conclusion, IV/PO MXF monotherapy is clinically and bacteriologically similar to PIP/TAZ–AMC in DFI and is an effective and valuable treatment option for patients with moderate to severe DFI. Moxifloxacin treatment was well tolerated; therefore, in combination with surgery, sequential MXF monotherapy is an appropriate treatment choice for DFI patients with a range of severities, particularly for those with polymicrobial infections. Although infection was cured in the majority of patients, many patients underwent surgery and/or an (initial) amputation, highlighting the importance of early recognition and subsequent prompt treatment of DFIs.

## Abbreviations

ABI: Ankle–brachial index
AE: Adverse event
AMC: Amoxicillin/clavulanic acid
b.d.: *Bis die*, twice daily
BMI: Body mass index
CI: Confidence interval
cSSSI: Complicated skin and skin structure infection
CRP: C-reactive protein
DFI: Diabetic foot infection
DRC: Data Review Committee
ECCMID: European Congress of Clinical Microbiology and Infectious Diseases
EOT: End of treatment
HbA_1c_: Glycosylated hemoglobin
ITT: Intent-to-treat
IV: Intravenous
MBV: Microbiologically valid
MRSA: Methicillin-resistant *Staphylococcus aureus*
MXF: Moxifloxacin
PCT: Procalcitonin
PEDIS: Perfusion, extent/size, depth/tissue loss, infection, and sensation
PIP/TAZ: Piperacillin/tazobactam
PP: Per-protocol
PO: *Per os*, orally
q.d.: *Quaque die*, once daily
t.d.s.: *Ter die sumendum*, three times daily
TOC: Test-of-cure
